# Oral leukoplakia and erythroplakia in young patients: a southern Brazilian multicenter study

**DOI:** 10.1590/1807-3107bor-2024.vol38.0069

**Published:** 2024-08-05

**Authors:** Alini Cardoso SOARES, Ana Paula Neutzling GOMES, Camila Barcellos CALDERIPE, Fernanda Gonçalves SALUM, Karen CHERUBINI, Manoela Domingues MARTINS, Lauren Frenzel SCHUCH, Laura Borges KIRSCHNICK, Lucas Guimarães ABREU, Alan Roger SANTOS-SILVA, Ana Carolina Uchoa VASCONCELOS

**Affiliations:** (a) Universidade Federal de Pelotas – UFPel, Dental School, Diagnostic Center for Oral Diseases, Pelotas, RS, Brazil.; (b) Universidade Estadual de Campinas – Unicamp, Piracicaba Dental School, Department of Oral Diagnosis, Piracicaba, SP, Brazil.; (c) Pontifícia Universidade Católica do Rio Grande do Sul – PUCRS, Division of Oral Medicine, Porto Alegre, RS, Brazil.; (d) Universidade Federal do Rio Grande do Sul – UFRGS, School of Dentistry, Department of Oral Pathology, Porto Alegre, RS, Brazil.; (e) Universidade Federal de Minas Gerais – UFMG, School of Dentistry, Department of Child and Adolescent Oral Health, Belo Horizonte, MG, Brazil.

**Keywords:** Leukoplakia

## Abstract

The objective of the present study was to investigate the frequency of oral leukoplakia and oral erythroplakia among young patients from three Brazilian reference centers in Oral and Maxillofacial Pathology. A retrospective study was carried out from 2011 to 2021 on 861 patients diagnosed with oral leukoplakia and oral erythroplakia. Demographic and clinicopathological data were evaluated. Fisher’s exact test was used to evaluate the association among sex, age, anatomical location, and histopathological diagnosis. A total of 83 (9.64%) cases involved young patients (aged <40 years). Among these, biopsy records were included in 31 (37.34%) cases, all of which received a clinical diagnosis of oral leukoplakia. Seventeen (54.84%) patients were female, mostly in their fourth decade of life (n = 22/70.97%), and their mean age at diagnosis was 32.61(± 5.21) years. Among informed cases, seven (22.58%) patients were smokers. The lateral border of the tongue (n = 9/29.03%) was the most affected site. In 13 (41.94%) cases, oral leukoplakias showed a homogeneous appearance. The mean size of the lesions was 1.47 cm (0.2–3.0 cm) and the mean time of disease progression was 64.37 (± 65.90) months. The histopathological analysis showed that 11 cases (35.48%) exhibited some degree of epithelial dysplasia. Acanthosis and/or hyperkeratosis were observed in 20 cases (64.52%). No significant associations were observed between sex and anatomical location, age and anatomical location, nor between sex and histological diagnosis (p > 0.05). Oral leukoplakia and oral erythroplakia are uncommon diseases in young patients. In this population, oral leukoplakia shows a slight predilection for women aged between 30 and 39 years.

## Introduction

Oral potentially malignant disorders (OPMDs) are clinical conditions involving the risk of cancer development, observed both in a clinically defined precursor lesion and in clinically normal mucosa.^1^ The WHO Collaborating Centre for Oral Cancer classified 11 oral disorders as OPMDs, including oral leukoplakia (OL) and oral erythroplakia (OE),^1^ and OL was the most frequent finding in clinical practice.^1^The worldwide prevalence of OL ranges between 2.60% and 4.11%, and males after the fourth decade of life are the most affected individuals.^2^

Clinically, the disease is classified according to appearance as homogeneous or non-homogeneous (speckled/erythroleukoplakia, nodular or verrucous).^1^OE is less frequent than OL and exhibits a higher risk for malignant transformation.^3-5^ It is estimated that the prevalence of OE varies between 0.02% and 0.83%, and the condition is predominantly observed in male adults between the sixth and seventh decades of life.^4^The rate of overall malignant transformation is 9.8% in OL and varies from 14% to 50% in OE.^4,6^

Most oral squamous cell carcinomas (OSCCs) are diagnosed between the fifth and sixth decades of life.^4^However, epidemiological studies have demonstrated an increased incidence of OSCCs among young patients, eventually accompanied by an increased incidence of OPMDs and/or an increased risk of malignant transformation in this population.^7^ To date, limited information about OL and OE in young patients has been published in the literature.^8-12^ Considering the importance of these diseases for public health, the objective of the present study was to evaluate the frequency and demographic and clinicopathological characteristics of OL and OE in young patients based on a retrospective analysis of cases diagnosed and treated at three Brazilian reference centers in Oral and Maxillofacial Pathology.

## Methods

### Study design, setting, and ethical issues

Records of patients with a clinical diagnosis of OL and OE were retrieved in a retrospective study of data obtained from 2011 to 2021. Data were obtained from a consortium of three services of Oral and Maxillofacial Pathology located in the southern Brazilian region: Federal University of Pelotas (UFPel), Federal University of Rio Grande do Sul (UFRGS), and Pontifical Catholic University of Rio Grande do Sul (PUCRS). The study was approved by an institutional Research Ethics Committee (process no. 62023922.0.1001.5317) and followed the Declaration of Helsinki guidelines.

### Sampling

The study followed the STROBE guidelines.^13^ The clinical diagnosis of OL and OE was established according to Warnakulasuriya et al.^1^Patients aged less than 40 years with a clinical diagnosis of OL or OE and with histological evaluation related to the clinical diagnosis were selected. OL and OE are clinical diseases that can receive a histological diagnosis of acanthosis, hyperkeratosis, and oral epithelial dysplasia. Patients with lesions located on the lips were excluded due to the distinct etiopathogenesis. Cases with histological features of OSCC were not included in the sample. Finally, records of patients lacking information about the histopathological diagnosis were also excluded.

### Data collection

When available, the following data were collected: patient’s age (in years), sex (male or female), habits (smoking and/or alcohol drinking), anatomical location (base of the tongue, dorsal tongue, lateral border of the tongue, floor of the mouth, buccal mucosa, gingiva, hard palate, soft palate, lip commissure, or multiple sites, the latter when in more than one location), appearance (homogeneous or non-homogeneous, for OL), size (in cm), time of disease progression (in months), and histopathological diagnosis (acanthosis and/or hyperkeratosis, oral epithelial dysplasia, carcinoma *in situ*).

Regarding habits, patients who had smoked more than 100 cigarettes in their lifetime and who had smoked at least once in the last 30 days were considered smokers.^14^ Regarding alcohol consumption, patients who drank about five or more alcoholic beverages (approximately 60 grams of ethanol) at least once a month were considered alcoholics.^15^

Oral epithelial dysplasia grading remains a controversial issue, as the assessment and classification of dysplasia can be highly subjective. The WHO classification of oral epithelial dysplasia considers 16 architectural features and 11 cytological features. The diagnostic categories were separated into three levels of dysplasia (mild, moderate, and severe) and the classification was carried out according to the number of affected thirds.^16^ According to the latest WHO classification, mild dysplasia can be defined by cytological atypia limited to the basal third, moderate dysplasia by atypia at the middle third, and severe dysplasia by atypia at the upper third.^16^ Cases that were originally diagnosed as carcinoma *in situ* were reclassified as severe dysplasia.^16^The cases were diagnosed by an oral and maxillofacial pathologist at their respective services.

### Statistical analysis

The statistical analysis was performed using the Statistical Package for the Social Sciences (SPSS) for Windows, version 25.0 (ISPSS Inc., Chicago, USA). Descriptive statistics were carried out to characterize the cases regarding the following information: patient’s sex, age and habits, anatomical location of the lesion, appearance, and histopathological diagnosis. Fisher’s exact test was used to evaluate the association among sex, age, anatomical location, and histopathological diagnosis.

## Results

**Figure 1:**
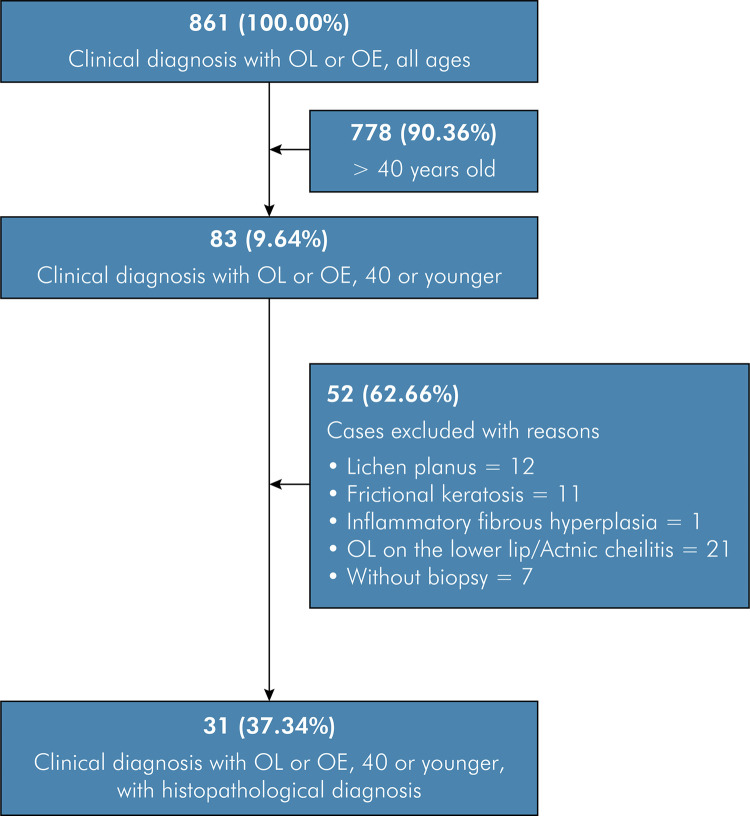
Flow diagram of the selection process.

**Figure 2:**
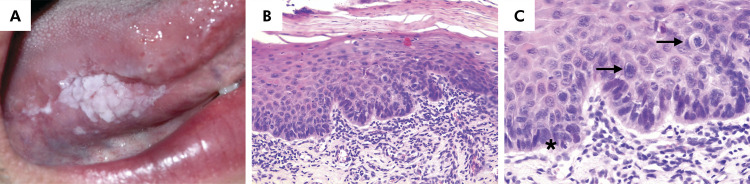
Oral leukoplakia. A, Non-flat, white plaque exhibiting sharp and well-defined borders in a 34-year-old man. B, Epithelium with a brightly eosinophilic keratin surface showing architectural and cytological changes of dysplasia (hematoxylin & eosin [H&E], 200×). C, Severe dysplasia showing loss of basal cell polarization, budding rete ridges, increased hyperchromasia (*), and mitotic figures (arrows) confined to the basal and parabasal layers (H&E, 400×).

Of the 861 patients diagnosed (clinical diagnosis) with OL or OE during the study period, 83 (9.64%) were 40 years old or younger. The remaining patients (n = 778/90.36%) were aged over 40 years. Among these 83 cases, 31 (37.34%) received histopathological diagnosis compatible with OL (clinical diagnosis) (Figure 1). No cases of OE (clinical diagnosis) were found in the 40-year-old or younger patients. The remaining patients (n = 52/62.66%) were excluded after the application of clinical and histopathological criteria. Seventeen (54.84%) patients were female and 14 (45.16%) were male (female-to-male ratio 1.2:1). Individuals in the fourth decade of life were the most affected (n = 22/70.97%). The mean age at clinical diagnosis was 32.61(± 5.21) years (range: 18 to 39 years). Data on habits were available in 12 cases, among which seven (22.58%) were smokers, three (9.67%) were nonsmokers or alcoholics, one (3.23%) was an alcoholic, and one (3.23%) was a smoker and an alcoholic. Regarding anatomical location, nine (29.03%) cases were on the lateral border of the tongue, eight (25.80%) in the buccal mucosa, and eight (25.80%) at multiple sites. Regarding clinical appearance, 13 (41.94%) cases were homogeneous and eight (25.80%) were non-homogeneous. The mean size of the lesions was 1.47 cm (range 0.2–3.0 cm). Information about time of disease progression was available in eight (25.81%) cases, with a mean time of 64.37 (± 65.9) months. The histopathological diagnosis included acanthosis and/or hyperkeratosis (n = 20/64.84%), mild dysplasia (n = 8/25.80%), moderate dysplasia (n=2/6.45%), or severe dysplasia (n = 1/3.23%) (Figure 2). The demographic and clinical data of the sample are displayed in Table 1. No statistical associations were observed between sex and anatomical location (p-value = 0.399), age and anatomical location (p-value = 0.112), or sex and histological diagnosis (p-value = 0.296). Data are displayed in Tables 2 and 3.

**Table 1:** Demographic and clinical characteristics of the sample.

Variable	n (%)
Sex (n = 31)	
Female	17 (54.84)
Male	14 (45.16)
Female-to-male ratio	1.8–2.2
Age (n = 31)	
Mean	32.61 ± 5.21
Range	18–39
Decades of life (n = 31)	
10–19	1 (3.23)
20–29	8 (25.80)
30–39	22 (70.97)
Habits (n = 31)	
Alcohol	1 (3.23)
Tobacco	7 (22.58)
Alcohol and tobacco	1 (3.23)
No alcohol and tobacco	3 (9.67)
Not informed	19 (61.29)
Anatomical location (n = 31)	
Dorsal tongue	1 (3.23)
Lateral tongue	9 (29.03)
Buccal mucosa	8 (25.80)
Gingiva	3 (9.68)
Soft palate	1 (3.23)
Lip commissure	1 (3.23)
Multiple sites	8 (25.80)
Appearance (n = 31)	
Homogeneous	13 (41.94)
Non-homogeneous	8 (25.80)
Not informed	10 (32.26)
Size (n = 23)	
Mean	1.47 (± 0.99)
Range	0,2-3,0
Time of disease progression (n = 8)	
Mean	64.37 (± 65.90)
Range	1–180
Histopathological diagnosis (n = 31)	
Acanthosis and/or hyperkeratosis	20 (64.52)
Mild dysplasia	8 (25.80)
Moderate dysplasia	2 (6.45)
Severe dysplasia	1 (3.23)

**Table 2:** Relation between age, sex, and anatomical location.

Age	10–19		20–29		30–39		Total
Anatomical location	♂	♀	♂	♀	♂	♀	♂ + ♀
Dorsal tongue	1	0	0	0	0	0	1
Lateral tongue	0	0	0	4	2	3	9
Buccal mucosa	0	0	1	0	3	4	8
Gingiva	0	0	0	1	1	1	3
Soft palate	0	0	0	1	0	0	1
Lip commissure	0	0	0	0	1	0	1
Multiple	0	0	1	0	4	3	8
**Total**	**1**	**0**	**2**	**6**	**11**	**11**	**31**

p-value (sex and anatomical location) = 0.399 – Fisher’s exact test/ p-value (age and anatomical location) = 0.112 – Fisher’s exact test.

**Table 3:** Relation between sex, location, and oral histological diagnosis.

Oral histological diagnosis	Acanthosis and/or hyperkeratosis		Mild dysplasia		Moderate dysplasia		Severe dysplasia		Total
Location	♂	♀	♂	♀	♂	♀	♂	♀	♂ + ♀
Dorsal tongue	0	0	1	0	0	0	0	0	1
Lateral tongue	1	5	0	1	0	0	1	0	8
Buccal mucosa	2	4	2	1	0	0	0	0	9
Gingiva	1	1	0	1	0	0	0	0	3
Soft palate	0	1	0	0	0	0	0	0	1
Lip commissure	0	0	1	0	0	0	0	0	1
Multiple	3	2	1	0	1	1	0	0	8
**Total**	**7**	**13**	**5**	**3**	**1**	**1**	**1**	**0**	**31**

p-value (sex and location) = 0.399/ p-value (sex and histological diagnosis) = 0.296.

## Discussion

The epidemiology and clinical profile of OL and OE have not been well documented in young patients. In the present study, 3.60% (31 cases) of 861 clinical diagnoses of OL involved young patients. As observed in our research, Azevedo et al.^17^ reported no OE cases in patients aged less than 40 years in a sample of 953 OPMDs, suggesting that cancerization is time-dependent. In a recent systematic review of the clinical and demographic characteristics of 1,246 individuals with OL, Roza et al.^7^observed that young patients comprised 9.23% of the sample (n = 115). In a Brazilian epidemiological survey of 107 OLs, a total of 30 (28.04%) cases involved patients aged 25 to 45 years.^18^ In a South African cross-sectional study of 95 patients with a clinical diagnosis of OL, Chandran et al.^8^ reported that 21 (22.11%) of them were individuals aged 20 to 39 years. In a Chinese study on the malignant transformation of oral epithelial dysplasia, only six (16.22%) out of 37 cases of OL were patients aged less than 40 years.^10^The literature emphasizes that OL is the most common OPMD, a distribution probably related to local cultural habits and different socioeconomic status among populations.^19^Also, studies reporting opposite results were mainly based on different sample sizes.^20,21^Finally, some researchers have shown that, in developed countries, OL tends to be diagnosed after the age of 40 years due to the use of samples recruited from hospitals rather than from the community.^20,21^

In our study, there was a slight predilection for female patients (n=17/54.84%), whereas Roza et al.^7^observed a predominance of males (87.8%) among young patients with OL. According to Mello et al.,^3^ most OPMDs occur in men, and the difference in distribution between sexes can be explained by cultural habits, especially tobacco use. Interestingly, the literature emphasizes that females, despite being less affected, exhibit a higher risk of malignant transformation of OLs, with an overall rate of 13.1%.^5,19^ However, it is unclear why women are more predisposed to malignant transformation compared to men. Some studies have already indicated that non-smoking women have an additional risk of malignant transformation, which can be explained by global genomic arrays that may illustrate a differential gene expression.^5,22^These young women with OL will possibly be the group with a growing number of oral cancer, as discussed by Toner and O’Regan,^23^ i.e., non-smoking young females aged <40 years. This profile of cancer patients seems to be increasing, and clinical and biological understanding remains minimal.^24^Conversely, a recent large multicenter study that assessed the frequency of OSCC in young patients showed that 5.8% (n = 626) of the patients were 40 years old or younger.^25^ Among them, 268 (42.8%) were women. These contrasting data suggest a need for future studies to explore the possible genetic role of sex in young patients with OL and OSCC.

The anatomical site, clinical appearance, and size of OLs are classical features that may influence the risk of malignant transformation of OPMDs.^5,26^A recently published systematic review demonstrated that the rate of malignant transformation is approximately 6.9% among young patients.^7^Lee et al.^9^showed that the relative risk for malignancy in leukoplakias on the tongue/floor of mouth was 28.13 times higher compared to malignancy on the buccal mucosa. The literature emphasizes that the ventral and lateral borders of the tongue and the floor of the mouth comprise the areas of overexposure to carcinogens as a result of the accumulation of saliva in alcohol and tobacco users.^5^Interestingly, eight cases in our sample had multiple locations. We carefully investigated whether proliferative verrucous leukoplakia (PVL) might be present in these patients. The leadership of the American Academy of Oral and Maxillofacial Pathology (AAOMP) and the North American Society of Head and Neck Pathologists (NASHNP) has recently approved a consensus guideline on PVL that was presented by a team of experts. The guideline stated that, “it is imperative to consider both the clinical presentation and history in concert with the histopathology of representative specimens in order to establish a PVL diagnosis”.^27^Due to the cross-sectional design of the study, sufficient information on the OLs was lacking, with consequent difficulty in identifying these multiple-site cases as PVL. Finally, it is important to consider that, as pointed out by Müller,^28^ some white lesions, such as frictional keratosis, are still misdiagnosed as OL.

In the present study, no statistical correlation was observed between anatomical site and the presence of oral dysplasia. Oral epithelial dysplasia is the most significant feature associated with the risk of malignant transformation to oral cancer.^29^In their study, Roza et al.^7^ noted that most cases did not have any degree of oral epithelial dysplasia (n = 73/64.6%), similar to what was observed in our sample (n = 20/64.52%).^7^ Chandran et al.^8^also reported the absence of epithelial dysplasia in 11 (52.38%) out of 21 young patients with OL. The results of different investigations concerning the relationship between epithelial dysplasias need to be interpreted with caution because the exercise of grading epithelial dysplasia is highly subjective, with low interpersonal reproducibility. Considering the slow process of oral carcinogenesis, it is important to emphasize the need for a periodic clinical evaluation of any OPMDs, considering their risk of malignant transformation.

It is a consensus in the literature that non-homogeneous OLs exhibit a higher risk of malignant transformation than homogeneous OLs.^1,3-20^In their systematic review of 24 retrospective surveys, Warnakulasuriya and Ariyawardana^19^detected a malignant transformation rate of 3% and 14.5% for homogeneous and non-homogeneous OL, respectively. Curiously, among our cases, no oral epithelial dysplasia was observed in five out of eight non-homogeneous OLs. OL lesions exceeding 200 mm^2^ are at increased risk for malignant transformation^19^ and most studies agree that malignant transformation of OPMDs is higher within the first 5 years after diagnosis.^5^ In the present study, three of the four cases that exceeded 200 mm^2^ received the histopathological diagnosis of acanthosis and/or hyperkeratosis. Unfortunately, no research evaluated size, time of disease progression or follow-up in young OL patients.

Tobacco use and alcohol consumption are well-established etiological factors for the development of OPMDs, and the literature states that the risk of progression of OSCC is directly related to these habits.^5^ Farquhar et al.^30^analyzed the risk and survival factors of oral tongue carcinoma among young patients and reported that, out of a total of 117 patients aged up to 45 years, 59 (50%) were female and less likely to use tobacco (n=59/51%) and alcohol (n = 102/90%).^30^Some authors have demonstrated increased genomic instability in young patients, suggesting the presence of genetic differences between young and older individuals affected by OSCC.^31^Moreover, unidentified etiological agents or even unknown risk factors for oral carcinogenesis should be considered in young people. Unfortunately, just a few studies specified information about tobacco and alcohol consumption, and the absence of standardization regarding the concepts of these habits did not allow for a more accurate interpretation.

The results of this research have limitations that should be addressed. First, the limited number of cases may not accurately represent the true frequency of OL in the Brazilian population. Second, some information was missing or lost over time due to the retrospective study design. The absence of electronic records in current Brazilian services and the lack of protocols used to describe patient details hinder data collection and subsequent evaluation. Clinical centers must find ways to implement standardized instruments in order to provide better data collection. Third, it was difficult to find works in the literature that classify OPMDs by age group, not allowing for data extraction. Finally, no studies evaluating the genetic profile of young patients with OPMDs were found in the English, Spanish, or Portuguese literature. Therefore, we emphasize the importance of well-designed prospective clinical studies for a better understanding of OL in young patients. In addition, journals should encourage the use of protocol guides (for example, STROBE in observational studies) in order to standardize the description of the clinical and demographic data of the patients.

## Conclusion

In summary, this multicenter study shows that OL and OE are uncommon lesions in young patients. In this population, OL shows a slight predilection for women aged 30 to 39 years. Considering the potential risk of malignant transformation of OPMDs, general dentists should be aware of all patients with suspicious oral lesions, regardless of their age.
